# Inducible but Not Constitutive Expression of PD-L1 in Human Melanoma Cells Is Dependent on Activation of NF-κB

**DOI:** 10.1371/journal.pone.0123410

**Published:** 2015-04-06

**Authors:** Kavitha Gowrishankar, Dilini Gunatilake, Stuart J. Gallagher, Jessamy Tiffen, Helen Rizos, Peter Hersey

**Affiliations:** 1 Melanoma Research, Kolling Institute of Medical Research, University of Sydney, Royal North Shore Hospital, St Leonards, Sydney, New South Wales, Australia; 2 Faculty of Medicine and Health Sciences, Macquarie University, Sydney, New South Wales, Australia; Texas A&M University, UNITED STATES

## Abstract

Monoclonal antibodies against immune checkpoint blockade have proven to be a major success in the treatment of melanoma. The programmed death receptor-1 ligand-1 (PD-L1) expression on melanoma cells is believed to have an inhibitory effect on T cell responses and to be an important escape mechanism from immune attack. Previous studies have shown that PD-L1 can be expressed constitutively or can be induced by IFN-γ secreted by infiltrating lymphocytes. In the present study we have investigated the mechanism underlying these two modes of PD-L1 expression in melanoma cells including cells that had acquired resistance to the BRAF inhibitor vemurafenib. PD-L1 expression was examined by flow cytometry and immunoblotting. Specific inhibitors and siRNA knockdown approaches were used to examine the roles of the RAF/ MEK, PI3K, NF-κB, STAT3 and AP1/ c-Jun pathways. IFN-γ inducible expression of PD-L1 was dependent on NF-κB as shown by inhibition with BMS-345541, an inhibitor of IκB and the BET protein inhibitor I-BET151, as well as by siRNA knockdown of NF-κB subunits. We were unable to implicate the BRAF/MEK pathway as major regulators in PD-L1 expression on vemurafenib resistant cells. Similarly the PI3K/AKT pathway and the transcription factors STAT3 and c-Jun had only minor roles in IFN-γ induced expression of PD-L1. The mechanism underlying constitutive expression remains unresolved. We suggest these results have significance in selection of treatments that can be used in combination with monoclonal antibodies against PD1, to enhance their effectiveness and to reduce inhibitory effects melanoma cells have against cytotoxic T cell activity.

## Introduction

The introduction of monoclonal antibodies (MAbs) that block the checkpoint receptor programmed death receptor (PD1) and its ligand (PD-L1/CD274/B7-H1) in the treatment of melanoma has been a major breakthrough in the treatment of this disease. The first report from treatment with the anti PD1 MAb nivolumab indicated that treatment was associated with overall response rates of 28% and median survivals of 24 months (1). One and 2 year survival rates were 62% and 43% respectively [1]. Treatment with a second MAb referred to as MK3475 (pembrolizumab) produced overall response rates of 38% that increased further at some dose schedules [2]. Even greater response rates and survivals were suggested by preliminary results from treatment of small patient groups with a combination of nivolumab and ipilimumab [3]. Response rates in patients treated concurrently with 1mg/kg of nivolumab and 3mg/kg of ipilimumab were 48% and 1 year survivals in the 17 patients treated were 81%. These results are now being evaluated in large randomised phase III trials.

PD-L1 is expressed on dendritic cells (DCs), macrophages, activated T and B cells and several non-hematopoietic cells including cancer cells. Under certain conditions, engagement of PD-L1 with the PD-1 receptor on T cells results in decreased effector T cell function and apoptosis of T cells [4–6]. Blockade of the PD-1 pathway has been shown to be effective in restoring T cell function and immune responses against melanoma and other cancers [7]. Phase 1 studies in melanoma have suggested that PD-L1 expression is strongly associated with responses to nivolumab [8, 9]. These results were also supported by large phase 1 studies with pembrolizumab which showed a strong association with responses by RECIST criteria and improved progression free survival [2]. PD-L1 expression on some tumors may result from oncogenic pathways such as PI3 kinase signalling in gliobastomas in association with PTEN deletion, ALK and STAT3 signalling in lymphomas or mutant EGFR activity in lung tumors [10–12]. The Interferon regulatory factor IRF-1 was shown to be required for PD-L1 expression in a lung cancer cell line [13]. Initial immunohistochemical studies on melanoma tissue showed a strong correlation between PD-L1 expression and the presence of tumor infiltrating lymphocytes (TILs) [14, 15]. Only a small percentage of melanoma appeared to have oncogene driven expression compared to that described for lung carcinoma. Moreover it was shown in the same studies that PD-L1 could be up-regulated on melanoma by exposure to interferon gamma (IFN-γ). These observations have given rise to the concept that PD-L1 expression on melanoma is largely an adaptive feedback response by melanoma cells to immune attack by TILs. This response limits the effectiveness of immune responses in eradicating melanoma [16]. The actual mechanism of up-regulation of PD-L1 by IFN-γ was not specifically defined. Additional interest in the expression of PD-L1 on melanoma was generated by the observation that PD-L1 was up-regulated on melanoma cells that had become resistant to treatment with selective BRAF V600 inhibitors [17]. The main pathway involved in this up-regulation was MAPK signalling through MEK but c-Jun and STAT3 proteins also appeared to be required for the expression of PD-L1.

In previous studies on melanoma isolated from patients failing treatment with vemurafenib we found that resistance to vemurafenib induced apoptosis was associated with up-regulation of NF-κB [18] [19]. NF-κB is a key transcription factor that is involved in inflammation and resistance to cell death and is known to be activated in some melanoma [20–22]. The NF-κB family consists of seven proteins but the prototypical NF-κB is a heterodimer of p65 RelA/p50 which is sequestered in the cytoplasm by association with the inhibitor IκB-alpha. Stimulatory signals received by the cell result in the activation of IκB kinases that phosphorylate IκB-alpha leading to its degradation by proteasomes. This allows RelA/p50 to translocate to the nucleus where it binds to kB sequences activating transcription in the canonical activation pathway [23, 24].

Pharmacological inhibitors of NF-κB include the IκB kinase inhibitor BMS-345541 which prevents phosphorylation of IκB and thereby entry of RELA/p50 into the nucleus [25]. We have recently reported that NF-κB activity can be regulated by a class of epigenetic regulators, the BET (bromodomain and extra terminal) proteins [26]. The BET proteins influence gene expression as they “read” the “codes” or modifications written on histones, serve as co-regulators and thus play important roles in epigenetic regulation [27]. The BET protein inhibitor I-BET151, was shown to be a potent inhibitor of NF-κB in LPS stimulated macrophages [28] and of NF-κB in melanoma by inhibiting the transcription of p50 [26].

In the present study we have examined the expression of PD-L1 in a panel of melanoma lines that had both IFN-γ inducible and constitutive expression of PD-L1. We included cell lines taken from patients before treatment with vemurafenib and after development of resistance to vemurafenib. We report that IFN-γ inducible expression was dependent on activation of NF-κB in the cell lines tested. The inducible expression of PD-L1 could be down regulated by pharmacological inhibitors of NF-κB signalling which may have complementary roles with existing inhibitors of PD1 in treatment of melanoma. We found minimal involvement of the BRAF/MEK pathway irrespective of whether the melanoma cells were resistant to vemurafenib or not.

## Methods and Materials

### Cell lines

Human melanoma cell lines Mel-RMu, SK-Mel28, Mel-JD and Me1007 have been described previously [29]. Cells were cultured in Dulbecco’s modified Eagle medium (DMEM) containing 10% fetal calf serum (FCS) (AusGeneX, Brisbane, Australia). In addition primary melanoma cell cultures were established as described previously [30] from patients entered into the Roche “BRIM II” phase II study of vemurafenib in patients who had failed previous treatment. The patient lines were established prior to and during relapse from treatment with vemurafenib, labelled “pre” and “post” respectively and as described elsewhere [31]. In this study we included those named Patient 1 pre and post, Patient 3 post and KMJR138. All cell lines (except SK-Mel28) were established previously in our laboratory from patient tumors and were tested for mutations using the OncoCARTA and or MelaCARTA panel and contained melanoma associated mutations. SK-Mel28 was originally purchased from ATCC and current batch confirmed using StemElite ID system (Promega, Madison, WI). In addition we also used the Geneprint 10 system (Promega, Madison, WI) for cell line authentications and matching.

### Ethics statement

These studies and cell lines generated were approved by the Hunter and New England Research Ethics Committee, Australia.

### Chemicals and reagents

I-BET151 was supplied by GlaxoSmithKline (Brentford, UK). BMS-345541, BKM120, BEZ235, GSK1120212-(Trametinib), GDC0973-(Cobemitinib) UO126, PLX4032 (Vemurafenib), GSK2118436 (Dabrafenib), LY294002, were purchased from Selleck Chemicals (Houston, TX, USA). IFN-γ was from Shenandoah Biotechnologies (Warwick, PA, USA).

### Flow Cytometry

Cells were treated with IFN-γ in the presence or absence of inhibitors and drugs I-BET151 or BMS-345541 for 48 hours, (or as indicated) harvested and washed with FACS buffer (1% FCS in PBS). They were stained for 30 min at 4°C with biotinylated anti-PD-L1, washed once in FACS buffer and incubated with secondary streptavidin-PE for an additional 30 min at 4°C. Cells were washed once and resuspended in FACS buffer and cell surface expression of PD-L1 was analysed by flow cytometry using FACS Calibur (Beckton Dickinson, Franklin Lakes, NJ, USA) and CellQuest Pro software. Live cells were gated using forward and side scatter. All PE fluorochrome detection was acquired in FL-2. Cells stained with mIgG2a-PE isotype were included in every experiment to set isotype values. Average PD-L1 expression levels are shown as mean fluorescence intensity (MFI) values and represented as a relative fold change over corresponding isotype control values. Streptavidin-PE, biotinylated-PDL1 and isotype controls were from Biolegend (San Diego, CA, USA). SD, SEM and average values were derived from three independent experiments unless otherwise specified and generated using GraphPad Prism (La Jolla, CA, USA). *** corresponds to a p-value of 0.0001; ** corresponds to a p-value of 0.001; and * corresponds to a p-value of 0.01.

### Western blotting

Western blot analysis and cell fractionation were carried out as described previously [32]. Labelled bands were detected by Immun-Star horseradish peroxidase chemiluminescence kit (Bio-Rad, Regents Park, NSW, Australia), and images were captured with the Fujifilm LAS-4000 image system (Fujifilm, Brookvale, NSW, Australia). Antibodies included in this study were against PD-L1 (Rand D, Minneapolis, MN, USA) p-ERK, t-ERK, cyclin D1, STAT3, p50/105, RelA/p65 (D14E15), actin, tubulin, PARP (Cell Signaling, Danvers, MA, USA); XIAP (20/hILP/X, BD) GAPDH, c-Jun, p-c-Jun and vinculin (Santa Cruz technologies, Dallas, TX, USA).

### siRNA transfections

For gene knock-down studies, the p65, and p50/p105 siRNAs were purchased from QIAGEN. Non-silencing control (1027281), and SMART Pool c-Jun silencers were purchased from Dharmacon (Millennium Science, VIC, Australia). Transfection was carried out using RNAiMax (Invitrogen/ Life technologies, Mulgrave, VIC, Australia) as previously described [33].

### Lentiviral transductions

Cells were transduced with either control or STAT3 silencers as previously described [34] with an MOI of 3 for 48 hours before they were treated with IFN-γ for an additional 48hours, and harvested for immunoblotting.

### q-RT-PCR

RNA was extracted from cell lines using RNeasy Plus mini prep kit (QIAGEN, Limburg, Netherlands), quantified using a Nanodrop (Thermo Scientific, Wilmington, DE) and 1μg RNA was reverse transcribed with SuperScriptIII (Invitrogen). The cDNA generated was used in the PCR reactions with the Universal PCR Master Mix and Taqman probes (Applied Biosystems) specific for PD-L1 [35] and normalized to levels of 18S RNA (Hs99999901_s1). q-RT-PCR was performed using the AB7900 (Applied Biosystems/Life technologies, Mulgrave, VIC, Australia).

### Analysis of cell death

Apoptotic cells were quantified using annexin-V staining as described by the manufacturer (Becton Dickinson, Franklin Lakes, NJ, CA), and measured using a Becton Dickinson FACS Calibur flow cytometer. Caspase inhibitor Q-VD-O-Ph was purchased from SM Biochemicals (Annaheim, CA, USA).

### NF-κB activity assays

Cells were transfected with the NF-κB Cignal reporter vector (CCS013L, QIAGEN), containing the firefly luciferase gene controlled by an NF-κB responsive promoter and a vector encoding the renilla gene under a constitutive promoter. After 24 hours, media was replaced with fresh media containing 10μM I-BET151 or vehicle control. After the indicated length of time, luciferase and renilla activity were detected using the Dual-Glo Luciferase Reporter kit as directed by the manufacture (Promega, Fitchburg, WI).

## Results

### IFN-γ induced PD-L1 expression in melanoma cells is time and dose dependent

**Fig 1:**
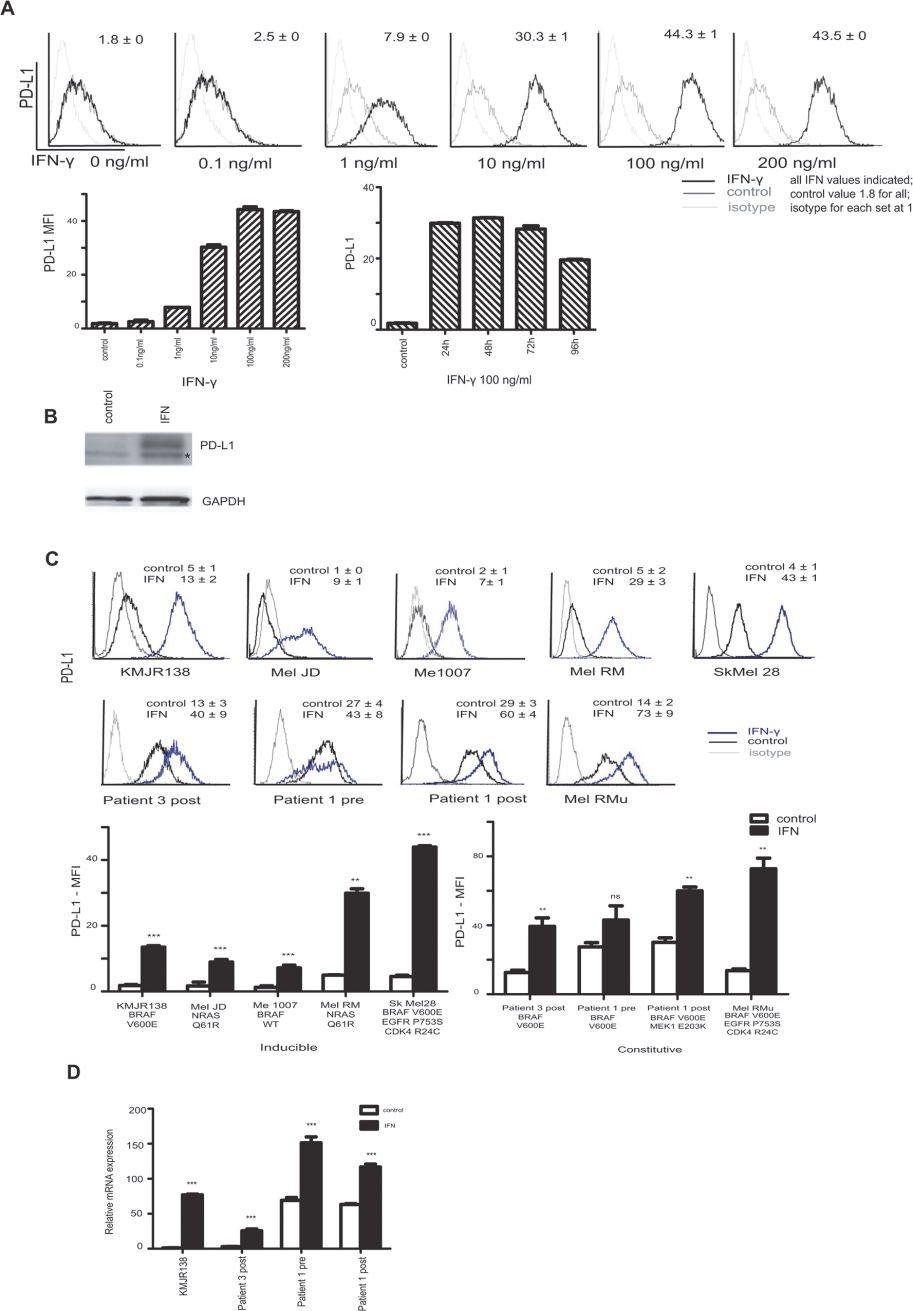
PD-L1 expression in melanoma cells. **A**. KMJR138 cells were treated with indicated doses of IFN-γ for 48 hours and the cell surface expression of PD-L1 was determined by flow cytometry. The corresponding mean fluorescence intensity (MFI) with values is shown in the histogram and plotted in the graph below and corresponds to the relative fold increase over isotype levels. The time course graph represents the MFI at the indicated times upon treatment with 100ng/ml IFN-γ. All values are an average of two independent replicates. **B**. PD-L1 protein detection induced after IFN-γ treatment for 48 hours is indicated in the representative immunoblot. * corresponds to a non-specific band. **C.** A panel of melanoma cell lines were treated with 100ng/ml IFN-γ for 48 hours and cell surface expression of PD-L1 was determined by flow cytometry. MFI is represented in the graphs (average of three independent replicates) and MFI values are indicated along with the histograms. Control refers to dimethyl sulfoxide (DMSO) treatment. **D.** Quantitative real time PCR was performed to evaluate induction of PD-L1 transcripts on RNA isolated from cells treated with DMSO (control) or 100ng/ml IFN-γ for 24 hours. Levels were normalised to the 18sRNA transcript. Values from one of two independent replicates are shown. ***p-value = 0.0001; **p-value = 0.001; *p-value = 0.01 ns = non-significant.

PD-L1 expression was examined by flow cytometry in a representative cell line established from a pre-vemurafenib treated biopsy (KMJR138) and is shown in Fig 1A. IFN gamma (IFN-γ) induced PD-L1 expression at concentrations as low as 1ng/ml, with significant induction at 10 and 100ng/ml. Sustained induction of PD-L1 for 96 hours was detected with concentrations of 100ng/ml. The mean fluorescence intensity (MFI) relative to isotype control levels is shown in the bar charts. PD-L1 was also detected by western blotting after 48 hours of IFN-γ treatment (Fig 1B). We investigated PD-L1 expression in a panel of melanoma cell lines which included Mel-JD, Mel RMu, Sk-Mel-28, Me1007, Mel-RM and patient derived cultures established before treatment and during relapse after treatment with vemurafenib (Patient 3 post and patient 1, pre and post—Fig 1C). Undetectable to very low levels of constitutive PD-L1 expression was seen in some cell lines (KMJR138, Me1007, Mel-RM, Mel-JD) whereas varying levels of constitutive expression of PD-L1 without the need for IFN-γ stimulation was detected in Patient 3 post, Patient 1 pre and post and Mel RMu cells. An expression level of at least fivefold higher than the isotype control was considered as constitutive expression of PD-L1. This level could be further increased in most cell lines upon induction with IFN-γ. Constitutively expressed PD-L1 varied from moderate (Patient 3 post and Mel RMu 5–15 fold) to high levels (Patient 1 >20fold). Among the cell lines that we tested, all the cell lines that displayed a constitutive expression pattern harbored a BRAF V600E mutation.

To determine whether the inducible expression in melanoma cells was regulated transcriptionally, quantitative real time PCR was carried out. As shown in Fig 1D, PD-L1 mRNA increased in the cells at 24 hours after addition of IFN-γ.

### IFN-γ inducible expression of PD-L1 can be decreased by using inhibitors of NF-κB (BMS-345541) and BET proteins (I-BET151)

**Fig 2:**
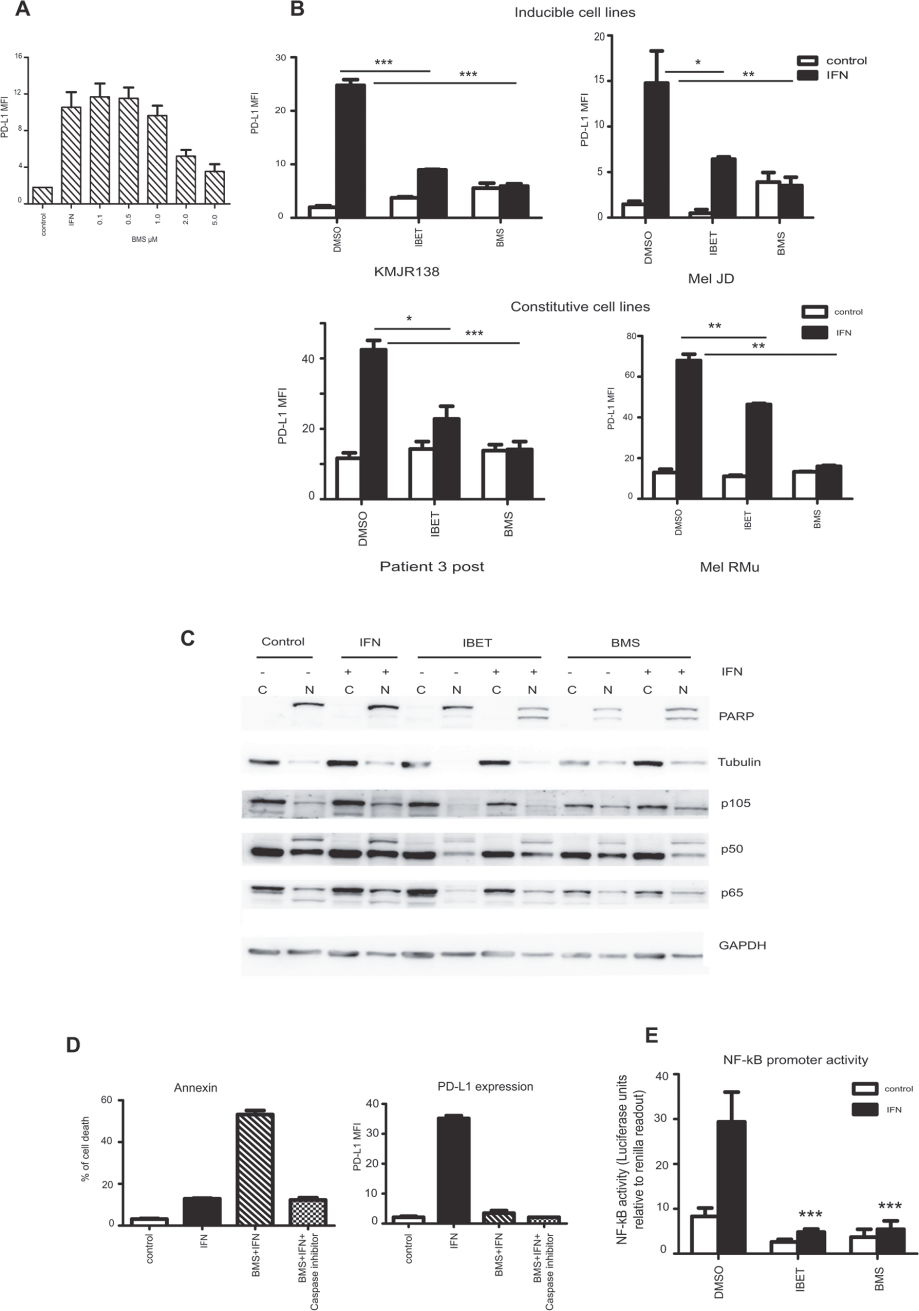
Targeting NF-κB reduces PD-L1. **A**. KMJR138 cells were treated with DMSO (control) or 100ng/ml IFN-γ (IFN) or indicated doses of BMS-345541 in the presence of IFN-γ for 48 hours before PD-L1 was analysed by flow cytometry. Mean Fluorescence Intensity (MFI) of PD-L1 expression is indicated; the average of two independent replicates is shown. **B**. Indicated cells were treated with either dimethyl sulfoxide (DMSO) (control) or 10μM of I-BET151 (IBET) or 5μM of BMS-345541 (BMS) for 48 hours in the absence or presence of 100ng/ml IFN-γ and PD-L1 cell surface expression was determined by flow cytometry. Mean fluorescence intensity (MFI) plotted is the average of three independent experiments. **C**. Sub-cellular fractionation of cellular lysates from a representative cell line (KMJR138) was probed for the indicated proteins. Cells were treated with DMSO (control), IFN-γ (IFN, +), I-BET151 (IBET), BMS-345541 (BMS) for 48 hours and separated into cytosolic (C) or nuclear (N) fractions. One representative blot out of two independent experiments is shown. **D**. KMJR138 cells treated with DMSO (control) or 100ng/ml IFN-γ, 5μM BMS-345541 or a combination of both for 48 hours in the absence or presence of 10μM pan-caspase inhibitor Q-VD-OPh were stained with Annexin V-APC, and PI to determine the number of apoptotic cells (left panel) and anti-PD-L1 antibody to determine the expression of PD-L1 (right panel) which is represented as the mean fluorescence intensity (MFI) over isotype controls. Average value from three independent experiments is indicated. **E.** Dual renilla-luciferase NF-κB reporter assay: KMJR138 cells were transfected with the control and NF-κB constructs for 24 hours and treated with the indicated inhibitors (10μM I-BET151 or 5μM BMS-345541) with or without 100ng/ml IFN-γ. Luciferase was measured and normalised to renilla values to determine promoter activity. Transfected cells were treated with the inhibitors for 24 hours and IFN-γ induction was carried out 1 hour prior to harvesting and recording luminescence. ***p-value = 0.0001; **p-value = 0.001; *p-value = 0.01. Average of two independent replicates experiments is indicated

To examine the role of NF-κB in the induction of PD-L1 we used the small molecule inhibitor BMS-345541 to reduce NF-κB activity. This inhibitor blocks the kinase activity of IKK, preventing the phosphorylation and degradation of IκB subunits, retaining NF-κB in the cytoplasm, preventing nuclear translocation and hindering its role in transcriptional activation [36]. In the inducible cell lines and in the moderately expressing cell lines, BMS-345541 was able to efficiently inhibit IFN-γ induction of PD-L1 even after 48 hours of treatment. Titration of BMS-345541 showed that 2μM and 5 μM were effective (Fig 2A and S1 Fig).

The small molecule inhibitor I-BET151 was shown by others [28] to inhibit NF-κB in LPS stimulated murine macrophages. We have also shown that inhibition of BET proteins can inhibit NF-κB in melanoma [26]. Here we show that I-BET151 (at the same doses i.e. 10μM) can efficiently decrease PD-L1 induction by IFN-γ in cell lines with little (KMJR138, Mel JD) or moderate levels of unstimulated PD-L1 expression (Patient 3 post, Mel RMu) (Fig 2B and S1 Fig).

Sub-cellular fractionation and analysis of the cellular lysates from the KMJR138 cells by immunoblotting revealed a decrease in p50 and p65 subunits in the nuclear fraction upon I-BET151 or BMS-345541 treatment in the presence of IFN-γ (Fig 2C). This correlated with a decrease in PD-L1 levels. PARP and tubulin were used as nuclear and cytoplasmic markers for fractionation. Inhibition of BET proteins and NF-κB were previously shown to induce apoptosis [26, 33].We confirmed that the reduction of PD-L1 levels was not merely an effect of induction of apoptosis by using the pan-specific caspase inhibitor Q-VD-OPh to block apoptosis (measured by Annexin-V staining). Upon BMS-345541 treatment, PD-L1 levels were reduced significantly even when the cells were prevented from undergoing cell death (Fig 2D).

Further evidence for the activation of NF-κB by IFN-γ was shown by the reporter assay in Fig 2E. Treatment of the KMJR138 cells with I-BET151 or BMS-345541 dramatically reduced the NF-κB promoter activity as early as 1 h (not shown) and was sustainable for over 24 hours. This corresponded to a decrease in PD-L1 similar to that shown in Fig 2A.

### IFN-γ inducible PD-L1 is decreased by siRNA mediated silencing of NF-κB and by transfection of a “NF-κB super repressor”

**Fig 3:**
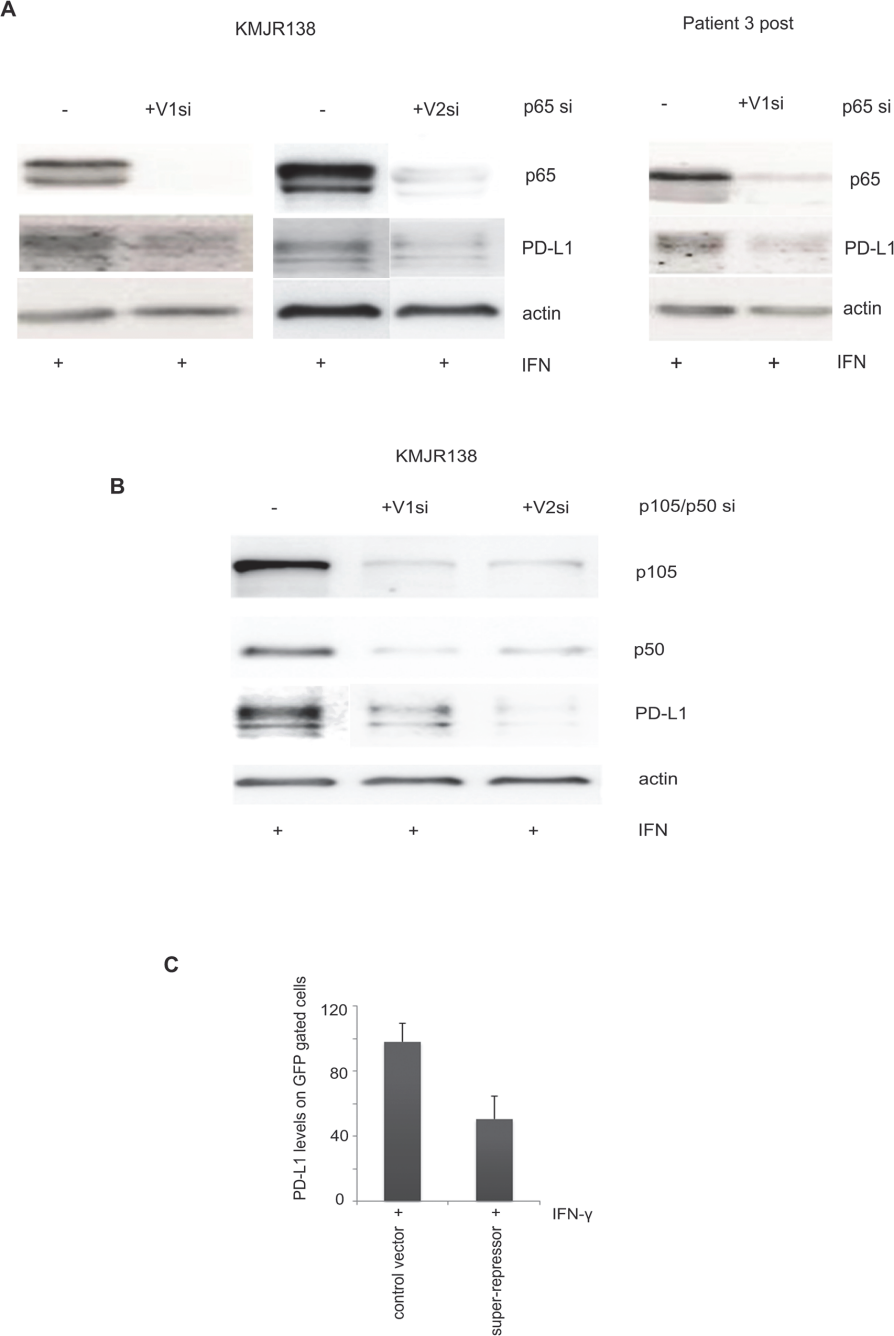
NF-κB is required for PD-L1 expression. **A**. Indicated cells were transfected with control si (-) or siRNAs against p65 (+) for 48 hours and treated with 100ng/ml IFN-γ for an additional 48 hours before they were harvested for immunoblotting for the indicated proteins. V2 si RNA transfection was done independently with the accompanying control siRNA. One representative out of three independent blots is shown. **B**. **C**.KMJR138 cells were transfected with either control (-) or two independent siRNA targeting p105/p50 (+) for 48 hours and treated with 100ng/ml IFN-γ for an additional 48 hours before being harvested for immunoblotting for the indicated proteins. One representative out of three independent blots is shown. **C.** Cells were transfected with either IκBwt-GFP (control vector) or IκBmutant-GFP (super-repressor) using lipofectamine 2000 for 48 hours after which 100ng/ml IFN-γ was added. PD-L1 expression was detected by flow cytometry on GFP gated cells and is represented as MFI; the average of two independent experiments is indicated. *p-value = 0.01.

We confirmed the role of NF-κB in PD-L1 expression by using siRNA mediated silencing of the sub units of NF-κB. Efficient knockdown of RelA/p65 with two independent silencer molecules resulted in a decrease in the IFN-γ induced expression of PD-L1 in KMJR138 (Fig 3A). Representative silencing in an additional cell line (Patient 3 post) is also shown. Additionally, two independent siRNA against p105/p50 were also transfected into cells and PD-L1 expression was analysed. A representative cell line is shown (Fig 3A). We also transfected melanoma cells with the wt IκB-GFP plasmid or the mutant IκB-GFP plasmid lacking the phosphorylation sites required for proteasomal degradation (mutation in S32 and S36). 48 hours after transfection the cells were treated with IFN-γ and 48 hours later PD-L1 expression was determined in GFP gated cells by flow cytometry. A decrease in expression of PD-L1 in the super-repressor transfected cells confirmed a requirement for NF-κB in the expression of PD-L1. Data from a representative cell line (Patient 3 post) is indicated in the Fig 3B.

### Role of STAT3 and c-Jun in PD-L1 expression

**Fig 4:**
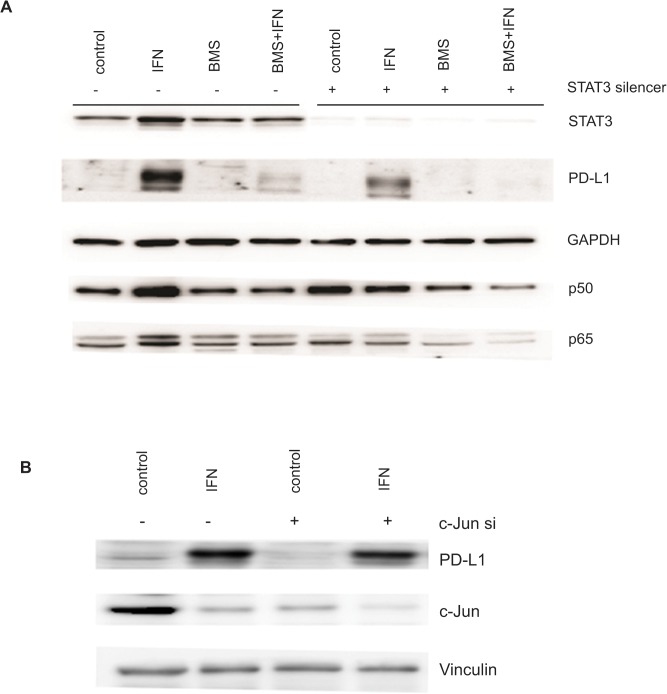
STAT3 and c-Jun independent expression of inducible PD-L1. **A**. KMJR138 cells were transduced using lentiviral constructs of either control pSIH-copGFP (-) or STAT3-copGFP (+) for 72 hours and treated with 5μM BMS-345541 (BMS) with or without 100ng/ml IFN-γ or DMSO (control) for an additional 48 hours. A representative immunoblot (out of three independent blots) for the indicated proteins is shown. **B**. KMJR138 cells were transfected with control siRNA (-) or with silencer (si) molecules against c-Jun for 48 hours and treated with DMSO (control) or IFN-γ (IFN) for an additional 48 hours and cellular lysates were immunoblotted for the indicated proteins. A representative blot out of two independent experiments is shown.

Previous studies have reported the requirement for the transcription factors STAT3 and c-Jun in PD-L1 expression [11, 17]. Silencing STAT3 has been implicated in increased phosphorylation of IκB leading to increased NF-κB activity. NF-κB in turn can activate STAT3 via IL-6. We used a lenti-viral based silencing strategy to knockdown STAT3 and siRNA based strategies to silence c-Jun followed by IFN-γ treatment to check the induced expression of PD-L1 (Fig 4). Despite efficient silencing of STAT3, PD-L1 was still induced by IFN-γ, albeit at a decreased level. This indicates that STAT3 plays a relatively minor role in PD-L1 expression. Similarly, c-Jun silencing did not have a significant impact on PD-L1 expression (Fig 4B). As shown in Fig 4A, p50 and p65 subunits of NF-κB were unchanged by knockdown of STAT3 indicating that it was not upstream of NF-κB and that regulation of PD-L1 by NF-κB was independent of STAT3. Data from a representative cell line is shown.

### IFN-γ inducible expression of PD-L1 is not abolished by inhibition of the MAPK or PI3K/AKT pathways

**Fig 5:**
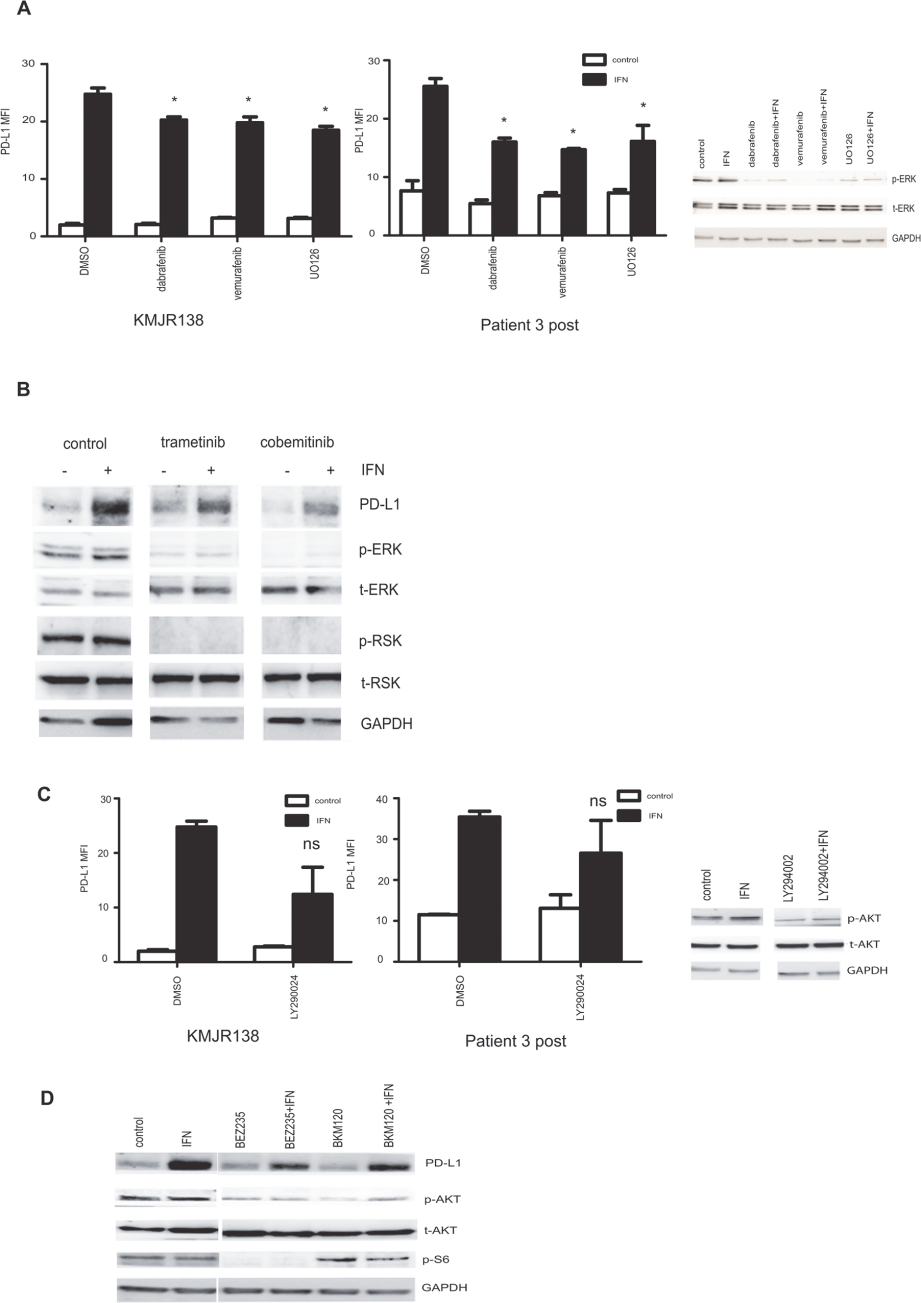
MAPK and PI3K inhibition are not effective in abolishing PD-L1 expression. **A**. Patient tumor derived cell lines KMJR138 or cell line from the Patient 3 post biopsy were treated with DMSO (control) or 10μM UO126 (MEKi) or 100nM dabrafenib (BRAFi) or 10μM vemurafenib (BRAFi) for 48 hours in the absence or presence of IFN-γ (+IFN) and PD-L1 expression was determined by flow cytometry. The mean fluorescence intensity (MFI) as a relative fold difference as compared to isotype controls is plotted. Average and SEM are derived from three independent experiments. KMJR138 cells were harvested 1 hour post treatment with the inhibitors and immunoblotted for the indicated proteins to detect decrease in p-ERK. **B**. Patient 3 post cells were treated with DMSO (control) or MEK inhibitors trametinib (100nM) and cobemitinib (1μM) and the immunoblot probed for PD-L1 and key proteins involved in the MAPK signalling pathway. Lanes have been depicted separately for clarity and are from the same representative blot. **C.** KMJR138 and patient 3 post cells were treated with 40μM PI3Ki LY294002 for 48 hours in the presence or absence of 100ng/ml IFN-γ (IFN) and PD-L1 expression was monitored using flow cytometry. An average of three independent experiments is indicated. The accompanying western blot indicates a decrease in p-AKT after treatment with LY294002. **D.** Patient 3 post cells were treated with DMSO (control), 100ng/ml IFN-γ (IFN) or 1μM BEZ235 or 1μM BKM120 for 24 hours and lysates were immunoblotted for the indicated proteins. *p-value = 0.01 ns = non-significant.

The MAPK signalling pathway is activated in the majority of melanoma cells. Previously, melanoma cells that have acquired resistance to BRAF inhibition were shown to have increased PD-L1 levels which could be decreased by treatment with the MEK inhibitor U0126 [17]. Melanoma lines that were relatively sensitive or resistant to vemurafenib (KMJR138 and patient 3 post respectively) were treated with MAPK inhibitors both in the presence and absence of IFN-γ. The KMJR138 cells showed minimal differences while the patient 3 post cells showed moderate differences in PD-L1 expression upon treatment with either BRAF or MEK inhibitors (Fig 5A). In addition, several cell lines were analysed after treatment with the MEK inhibitor, UO126 (S2 Fig) and confirmed minimal changes in induced PD-L1 expression. This was in contrast to what was observed when the cells were treated with NF-κB inhibitors as seen previously in Figs 2 and 3 and S1 Fig p-ERK levels were decreased as expected upon treatment with the MAPK inhibitors (Fig 5A). Additionally, western blot studies of cells treated with two MEK inhibitors that are in clinical use, (trametinib and cobemitinib) (Fig 5B) confirmed that the downstream effectors of the MAPK pathway remained inhibited but PD-L1 expression remained unchanged. A representative cell line from patient 3 is shown in Fig 5B.

To determine whether PI3kinase-AKT signalling was required for induction of PD-L1 expression the melanoma cells were treated with inhibitors of PI3K (LY294002 and BKM120) and with a dual inhibitor of PI3K and mTOR (BEZ235). A study on a representative cell line from Patient 3 which is known to express activated AKT is shown in Fig 5C. Despite a decrease in p-AKT and S6 levels indicated in the western blots shown in Fig 5D the expression of PD-L1 was not abolished.

### High constitutive expression of PD-L1 was not decreased by inhibition of NF-κB, MAPK and PI3 Kinase pathways

**Fig 6:**
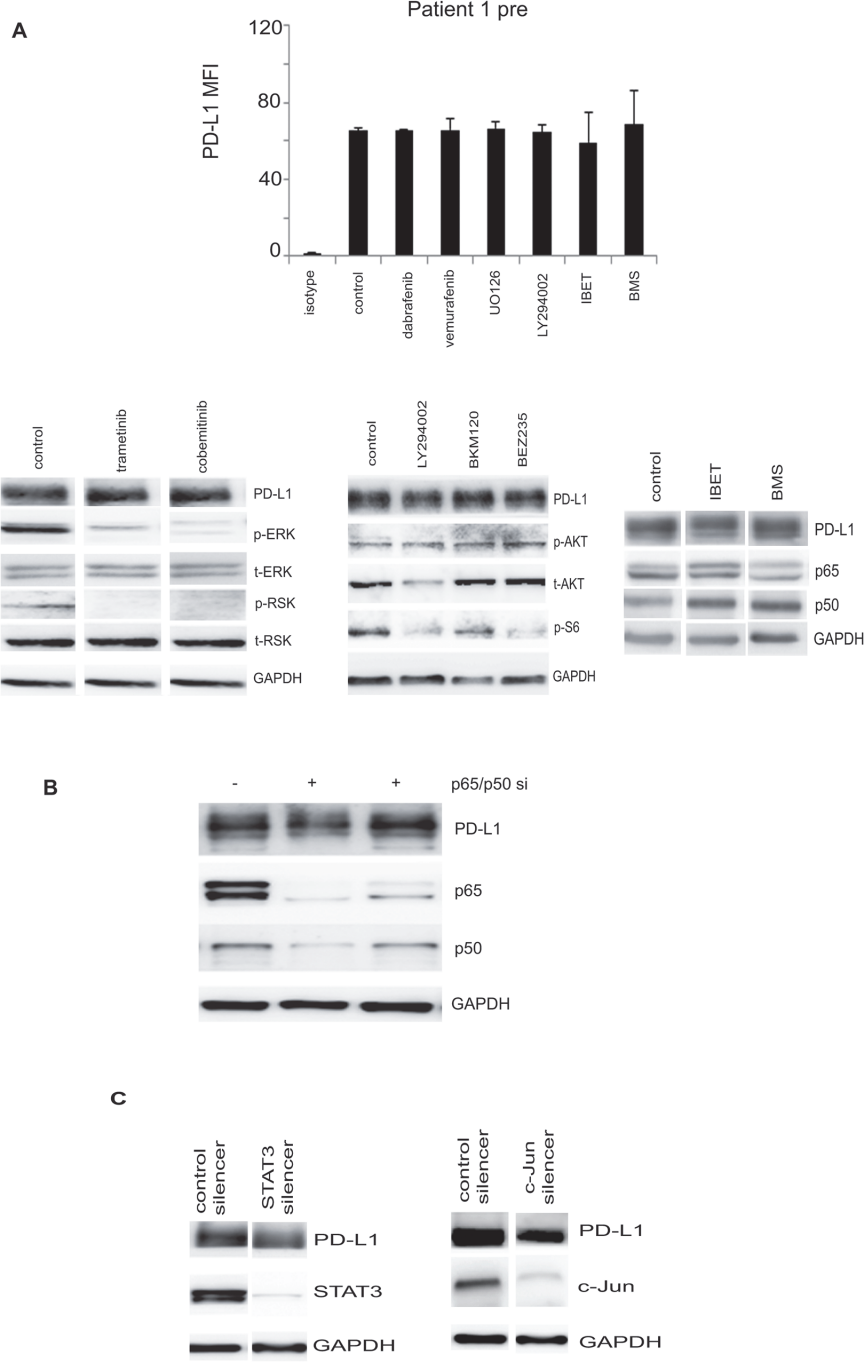
Effect of signaling pathway inhibition in cells expressing high levels of PD-L1. **A**. Patient 1 pre cells were treated with DMSO (control) or BRAF inhibitors (dabrafenib 100nM/ml or vemurafenib 10μM/ml) or 10μM of MEK inhibitor UO126 or 40μM PI3K inhibitor LY29004 for 48 hours and the average PD-L1 expression determined by flow cytometry is represented as a fold difference of the mean fluorescence intensity compared to isotype levels. The plotted average mean fluorescence intensity and SEM are derived from three independent experiments. Western blots for indicated proteins after treatment for 24 hours with trametinib or cobemitinib, LY290024, BKM120 and BEZ235 are also shown. Lanes were from the same blot and a representative blot out of two independent experiments is shown. Patient 1 pre cells were further treated with 10μM I-BET151 (IBET) or 5μM BMS-345541 (BMS) for 48 hours and PD-L1 expression determined by western blotting. **B.** Patient 1 pre cells were transfected with siRNA against the p65 and p50 subunits of NF-κB or control silencer (-) for 72 hours. Cellular lysates were immunoblotted for the indicated proteins. One representative blot out of three independent experiments is indicated. **C**. Cells were transduced using lentiviral constructs of either control pSIH-copGFP or STAT3-copGFP for 72 hours. Immunoblot for the indicated proteins is shown. Cells were also independently transfected with the control or SMART Pool c-Jun silencer for 48 hours and the cellular lysates were immunoblotted for the indicated proteins. One representative blot out of two independent experiments is indicated.

We analysed PD-L1 expression in the matched cell lines (BRAF V600E lines) from Patient 1 which had high constitutive expression of PD-L1. Treating the cells with either MAPK or PI3K inhibitors did not decrease the PD-L1 levels (Fig 6A and S3A Fig). Specifically, we treated the patient 1 pre cells with BRAF inhibitors, dabrafenib, vemurafenib or the MEK inhibitors UO126, trametinib or cobemitinib, or PI3 kinase/AKT inhibitors LY294002, BKM120 or BEZ235. As shown in Fig 6A and S3A Fig expected reduction in activation of proteins of the AKT pathway was detected with little effect on expression of PD-L1. Similarly, treatment with I-BET151 or BMS-345541 did not significantly decrease PD-L1 expression. Silencing NF-κB subunits also did not show a significant reduction of PD-L1 levels (Fig 6B) and STAT3 or c-Jun silencing did not down regulate PD-L1 expression in these cells (Fig 6C). Similar data was obtained with the constitutive expression in the patient 1 post cells (S3 Fig).

## Discussion

The inhibitory function of PD-L1 expression in melanoma is believed to be an important escape mechanism from immune attack. Previous studies in other cancers have shown that PD-L1 may either be induced by oncogenic signals leading to constitutive widespread expression on cancer cells as seen in lung cancer or can be induced as an adaptive response to IFN-γ released by T effector cells during their interaction with melanoma cells [15, 17]. The latter is the main form of expression in melanoma [14] and raises the possibility that better understanding of inducible expression in melanoma may lead to pharmacological treatments that complement existing treatments based on blockade by MAbs such as nivolumab or pembrolizumab.

In the current study we show that IFN-γ inducible expression of PD-L1 on melanoma cells correlated with NF-κB activity as measured by promoter reporter assays, western blots and flow cytometry. We further show that siRNA knockdown of the p50 and p65 subunits or addition of the small molecule inhibitors of NF-κB, BMS-345541 and the BET protein inhibitor resulted in inhibition of IFN-γ induced PD-L1 expression. These results are the first to implicate activation of NF-κB in the expression of PD-L1 in melanoma but are consistent with suggestions from previous studies on dermal fibroblasts and cell lines taken from patients with myelodysplastic syndromes where NF-κB was shown to be involved in TNF-α and IFN-γ inducible expression of PD-L1. NF-κB binding sites were also found in the PD-L1 promoter in human monocytes after treatment with LPS [37]. Co-culture of hepatocarcinoma cells with macrophages resulted in increased PD-L1 expression that was down regulated by inhibition of NF-κB and STAT3 [38].

The present studies extend these findings by showing that PD-L1 is regulated by NF-κB in melanoma and that it is only the inducible form of PD-L1 expression in melanoma that is regulated by NF-κB and not the constitutive forms of PD-L1 expression. The pathways by which IFN-γ regulates NF-κB in melanoma remain to be defined. Signalling from the IFN-γ receptor is complex and classically involves binding of Janus tyrosine kinases (JAKS) and activation of signal transducer and activation of transcription (STATs) factors like STAT1 which on entry into the nucleus activate a number of other transcription factors [39]. Nevertheless direct entry of the IFN-γ receptor into the nucleus has been described and would be consistent with the relatively early activation of NF-κB by 1 hour detected in the present studies (studies not shown). Given the duration of NF-κB activation over several days it is possible that additional secondary factors were involved in maintenance of NF- κB activation such as cytokine production resulting from activation of NF-κB. We examined whether several signalling pathways described in previous studies may be involved in IFN-γ inducible expression. In particular acquisition of resistance of melanoma cells to BRAF inhibition was found to be associated with up-regulation of PD-L1 [11] that was dependent on the MEK and PI3K pathways and activation of c-Jun and STAT3. Our studies on eight cell lines taken from patients before and after failing treatment with vemurafenib found only minimal involvement of these pathways but this does not exclude that these pathways may be involved in other cell lines. Both resistant lines showed constitutive expression of PD-L1 that could be further increased to a small extent with IFN-γ treatment. The constitutive components of the increased expression were not down regulated by inhibition of the MEK or PI3K pathways. These findings are similar to those reported in other studies on melanoma cell lines resistant to BRAF inhibition [40].

STAT3 involvement in up-regulation of PD-L1 has been reported in a number of studies and there is evidence for cross talk between NF-κB and STAT3 [41, 42]. In the present study, however pharmacological inhibition of STAT3 had relatively small effects on PD-L1 expression. Knockdown of STAT3 did not abolish inducible PD-L1. A role for STAT3 however cannot be entirely excluded as there was a small decrease in PD-L1 levels in the STAT3 knockdowns. It was notable that knockdown of STAT3 did not inhibit the effect of NF-κB inhibitors on PD-L1 expression showing that the NF-κB effect was independent of STAT3. Similarly, knockdown of c-Jun had minimal effects on inducible PD-L1 expression.

The mechanism underlying the constitutive expression seen in our cell lines remains the subject of further investigation. Targeting MAPK, PI3K, NF-κB, STAT3 or c-Jun did not decrease PD-L1 levels indicating that other signalling mechanismsmay be involved.A recent report on large numbers of melanoma cell lines revealed that there was no association between BRAF V600E and NRAS mutational status and PD-L1 expression [40].Constitutive expression in lymphomas was reported to be dependent on the ALK and STAT3 axis. In certain glioblastomas, the expression of PD-L1 was increased by PTEN deletion and PI3 kinase signalling [10]. EGFR signalling was shown to be involved in PD-L1 expression in non-small cell lung cancer [43] and pharmacological blockade using tyrosine kinase inhibitors of EGFR showed significant reduction in mouse lung cancer model systems [12]. The cell lines in the present study do not harbor activating EGFR mutations, so it is unlikely that PD-L1 expression is mediated by constitutive EGFR signalling.

The role of NF-κB in regulation of PD-L1 expression has not previously been appreciated and it remains to be established whether these results may have significance in developing combination treatments with MAbs against PD1 based on inhibition of NF-κB and whether subsets of patients with high levels of activated NF-κB may define patients not responding to PD1 checkpoint inhibitors.

## Supporting Information

S1 Fig Targeting NF-kB reduces PD-L1 in several melanoma cells. Cellular lysates from the inducible KMJR138 and Mel JD cell lines and constitutive Patient 3 post and Mel RMu cell lines were immunoblotted and probed for the indicated proteins. Cells were treated with either 10μM of I-BET-151 (IBET) or 5μM of BMS-345541 (BMS) for 48 hours with DMSO (control) or 100ng/ml IFN-γ. One representative blot for each cell line out of two independent experiments is shown.
(EPS)

S2 Fig Targeting MEK does not abolish PD-L1 in several melanoma cells. KMJR138, SkMel28 and Mel JD (inducible) and Patient 3 post and Mel RMu cell lines (constitutive) were treated with 10μM UO126 (MEKi) for 48 hours with DMSO (control) or 100ng/ml IFN-γ. Cellular lysates were immunoblotted and probed for the indicated proteins. One representative blot from two independent experiments is shown for each cell line.
(EPS)

S3 Fig Constitutive expression of PD-L1 in an independent cell line derived from Patient 1 post biopsy tumor. **A.** Patient 1 post cells were treated with DMSO (control) or BRAF inhibitors dabrafenib (100nM) or vemurafenib (10μM), or 10μM of MEK inhibitor UO126 or 40μM PI3K inhibitor LY29004 and the average PD-L1 expression was determined by flow cytometry. It is represented as a fold difference of the mean fluorescence intensity compared to isotype levels. The average value from three independent replicates is plotted. **B**. Patient 1 post cells were transfected with the control (-) or p65 silencers and independently with control or SMART Pool c-Jun silencer for 48 hours and blotted for the indicated proteins. Independently the cells were treated with DMSO (control), IFN-γ (IFN), 10μM I-BET151 (IBET) or 5μM BMS-345541 (BMS) in the absence or presence of 100ng/ml IFN-γ for 48 hours. All cellular lysates were immunoblotted for the proteins indicated. One representative blot from two independent replicates is show for each.
(EPS)
